# From concept to impact: strategic guidelines for environmental behavior change interventions

**DOI:** 10.3389/fpsyg.2024.1392461

**Published:** 2024-12-11

**Authors:** Mehmet Efe Biresselioglu, Muhittin Hakan Demir

**Affiliations:** Sustainable Energy and Climate Policies Research Centre (SENLAB), Izmir University of Economics, Izmir, Türkiye

**Keywords:** intervention, guideline, behavioral change, monitoring, ethics

## Abstract

Interventions aiming at behavioral change are common tools for assessing and stimulating environmentally friendly lifestyles. To obtain representative and scalable results from the interventions, the experimental design of the interventions is crucial. Likewise, an operational plan is significant concerning the coherence and consistency of the interventions and the comparability of the results from different interventions. Such a guideline contributes to the design, execution, and supervision of the interventions, provides standardisation and enhances collaboration with the intervention partners. Intervention guidelines also pertain to the strategic planning of data requirements and collection procedures. Based on the work conducted within the context of the EU-funded ENCHANT project, the guidelines presented in this manuscript are structured based on the key phases of planning and design, implementation, and analysis and reporting. Another key area that needs to be considered and included in the guidelines is the planning necessities for the administrative structure for the interventions. This includes allocating staff roles and responsibilities and potential challenges and obstacles that may arise during implementation. Ethical concerns are also addressed.

## 1 Introduction

The climate crisis becomes more visible through increasing greenhouse gas emissions, global warming, rising sea levels, and extreme weather events (IPCC, 2021). Climate change and environmental issues are closely related with human behavior such as the extensive use of fossil fuels (Gatersleben et al., 2023). Hence, individuals and households are significant in terms of combating the effects of climate change (Rau et al., 2022). However, it is not a straightforward task to motivate individuals towards pro-environmental behaviors (van Valkengoed et al., 2022). To this end, in recent years, the European Commission has significantly emphasised reducing carbon emissions from energy production and increasing energy efficiency in European lifestyles and economies. The European Climate Pact [European Commission (EC), 2021] sets ambitious goals, making energy transition a central focus, emphasising household-level energy efficiency improvements. Research and projects concerning behavioral change aim to increase the practices of energy efficiency among individuals and the public. Such endeavours utilise interventions to reach a high number of citizens. These interventions are critical in addressing negative behaviors and transforming them towards pro-environmental behaviors (Michie et al., 2011; Castán Broto and Bulkeley, 2013). These interventions can also be utilized as part of the proactive actions for climate change mitigation (Castán Broto and Bulkeley, 2013). Research on behavioral interventions is mainly focused towards identifying which interventions ‘work’ for which settings in order to encourage pro-environmental behavior and mitigate climate change. That is, such research seeks to determine the most effective intervention types (van Valkengoed et al., 2022). However, the effectiveness of interventions vary from one study to the other (Abrahamse and Steg, 2013).

A criticism of the behavioral interventions is that they are usually not based on well-defined methods (Rau et al., 2022). This calls for a more rigorous research design, along with well-defined guidelines to enhance causal inference. The existence of and adherence to such guidelines contribute to the reliability of the findings, replicability of the interventions, for instance, via reporting. Guidelines also foster a better understanding of the underlying processes, moderators, and mediators of the intervention (Gatersleben et al., 2023). Ehret et al. (2021) emphasize that the variety of implementation and evaluation methodologies for interventions make it difficult to perform a meta-analysis. Therefore, using more standard guidelines for interventions also contribute to the consistency in approaches, cross-comparability, and a larger base of effective interventions (Staddon et al., 2016). Planning interventions also enhances the proper allocation of resources and reducing the costs of the interventions (Gatersleben et al., 2023).

The experimental design of the interventions is crucial to obtaining representative and scalable results from the interventions. Likewise, an operational plan is significant concerning the coherence and consistency of the interventions and the comparability of the results from different interventions. Such a guideline reflects the design, execution, and supervision of the interventions, provides standardisation and enhances collaboration with the intervention partners. Intervention guidelines also pertain to the strategic planning of data requirements and collection procedures. The spectrum of intervention types, geographical, cultural, and organisational coverage of interventions makes it more crucial to identify a set of guidelines for implementation and monitoring of the interventions.

One key area that needs to be considered and included in the guidelines is the planning necessities for the administrative structure for the interventions. This includes allocating staff roles and responsibilities and potential challenges and obstacles that may arise during implementation. Ethical concerns are also addressed.

One crucial and complementary facet of the interventions is the meetings that enhance the collaboration between the various stakeholders of the interventions. Even though the theoretical design of the interventions may be designed with the utmost care, considering the relevant parameters, successful implementation of the interventions requires continuous monitoring and fine-tuning for configuring in terms of technical compatibility, alignment with geographical considerations, the availability and accessibility of communication channels, the potential to achieve the desired impact, and the needs of the target population. Hence, meetings for continuous monitoring and fine-tuning help to ensure smoother implementation and a more significant impact. With this perspective, interventions also serve to pinpoint the precise conditions under which intervention designs can be implemented successfully and effectively.

Another dimension concerning the stakeholders involved in implementing the interventions including energy providers and producers, government energy agencies and local administrations, and NGOs is the need to exploit their communication channels to connect with the target populations and implement interventions to foster alterations in energy-related behaviors. The multitude of these communication channels, advertising campaigns, web portals, SMS notifications, and smartphone apps emphasise the need to establish a unifying framework for implementing interventions that will be valid across all communication channels for enhancing the standardisation of the implementation and analysis.

The guidelines depicted in this manuscript are developed for the EU-funded ENCHANT Project (n.d.). ENCHANT has three principal objectives including: shifting the energy behavior of European households toward sustainability under real-world conditions, evaluating and systematising existing theoretical models, empirical data, and best practices related to psychological interventions that drive more sustainable energy choices, and exploring psychological, social, and contextual drivers of energy choices and sustainable energy lifestyles in European society. The project has utilised practical interventions for assessing how these factors can be effectively addressed in an implementable, replicable, and scalable setting at European, national, and local levels. The overall objective of the ENCHANT project is to support the energy transition by testing the impact of interventions affecting energy consumption behavior on a large scale across Europe. For this purpose, the project collaborated with user-partners which are energy companies, municipalities, and environmental NGOs in seven European countries. ENCHANT has tested established science-based behavioral intervention techniques in real-life settings in six countries (Norway, Italy, Romania, Türkiye, Austria and Germany) (ENCHANT Project, n.d.). The main results of the ENCHANT project include a policy matrix that demonstrates the strengths and potential weaknesses of interventions and offers behavioral strategies for practical implementation (ENCHANT Project, n.d.). The ENCHANT project has also developed the EnergyWizard tool that utilises the machine-learning with the projects’ findings for identifying the best intervention or a best combination of interventions along with the suitable communication channel for a group of people (ENCHANT Project, n.d.).

The ENCHANT project has started with developing intervention packages through an extensive review of theoretical models, empirical data, and best practices of psychological interventions designed to influence human behavior in the context of sustainable energy choices. Such analysis provided pointers for the design of interventions with the potential for scaling up. The next step was to establish procedures and protocols for the standardization of the interventions and the analysis.

The intervention development and implementation details are specified in the guidelines, application principles, and an operational implementation plan. As discussed above, such guidelines and operational plans are needed to ensure a successful implementation, provide standardisation, identify points of deviation from the plan, and formulate solutions to recover the intervention progress according to the plan.

The stakeholders involved in the interventions are significant in affecting the implementation and results of the interventions. The skills, competencies, capacities, capabilities, and resources of the intervention partners are significant drivers of the performance of the interventions. The ENCHANT project has collaborated with three key stakeholders to design and implement the interventions, focusing on specific groups and target populations. The first type of main stakeholders are energy providers and producers in the electricity and gas sectors. These stakeholders mainly participate in interventions concerning feedback on consumption, the provision of information, and the influence of social norms on the energy consumption patterns of individuals and households. The second type of stakeholders is the government energy agencies and local administrations such as municipalities. These stakeholders contribute mainly to interventions based on information dissemination, encouraging commitment, or organising energy-saving competitions to promote more sustainable energy behaviors among the broader public. The final type of stakeholders is the Non-Governmental Organizations (NGOs). NGOs operating in the energy and sustainability sector enhance the engagement of specific stakeholders and organised groups within civil society and the general public and contribute to the design and evaluation of interventions that rely on strategies like commitment-building, the dissemination of information, or influencing social norms, advocating for a sustainable energy transition and the adoption of sustainable lifestyles.

During the design and planning of the interventions, the data to be collected plays an essential role for measuring the impacts across various categories, such as policy support, public awareness, outreach, household interventions, primary energy savings, greenhouse gas emissions reduction, investments, and additional impacts. For this purpose, Key Performance Indicators (KPIs) are designed to specify how the performance of the interventions will be measured, and which data needs to be collected for these measurements.

The knowledge and experience gained from the interventions are also utilised to identify policy instruments that can be implemented and enablers and barriers in implementation and explore ways to enhance the potential for enablers to overcome barriers in real-life situations.

The guidelines for the interventions of the ENCHANT project were designed utilizing various systematic frameworks (Sundnes, 2014a; Sundnes, 2014b; Sundnes, 2014c; Hales et al., 2016). Other frameworks for interventions have also been developed. However, these frameworks mainly pertain to the research design (e.g., MINDSPACE), linking interventions to target behaviors (e.g., the behavioral change wheel), or limited to the public policy domain (e.g., BASIC) (Dolan et al., 2012; Michie et al., 2011; OECD, 2019).

The main types of interventions implemented by the ENCHANT project are feedback on own consumption, social norms, information provision including simplification, monetary incentives, commitment, competition, and collective and individual framing.

Within the context of energy-related behavior, feedback on own consumption involves informing individuals about their energy usage patterns and encouraging behavior change based on this information. Feedback interventions are effective via reflection on the outcomes of one’s behavior but are dependent on the individual’s awareness (Hayes and Cone, 1981; Bekker et al., 2010; Petersen et al., 2007; Xu et al., 2021; Shen et al., 2020a; Legault et al., 2020; Shen et al., 2020b; Canale et al., 2023).

Social norms, on the other hand, involve providing individuals with information about the energy consumption behavior of others and the socially acceptable standards of energy-related behavior, eliciting their approval or disapproval of specific behaviors. This type of interventions has the advantage of utilizing norms for behavior change. However, the efficiency is dependent on the individual’s perception of norms (Schultz et al., 2007; Graziano and Gillingham, 2015; and Barth et al., 2016; de Groot et al., 2021; Hewitt et al., 2023; Helferich et al., 2023; Belaïd and Flambard, 2023).

Information-provision based interventions offer individuals targeted data, providing information on, for instance, the environmental impact of one’s activities to encourage people to embrace energy conservation behaviors. Information provision is one of the most important prerequisites of behavior change. On the other hand, the effectiveness of this type of intervention is sensitive to the communication channel used (Komatsu and Nishio, 2015; Aydin et al., 2018; Asmare et al., 2021; Mundaca et al., 2023; Liu et al., 2023).

Monetary incentives refer to identifying how financial savings can motivate energy-related behaviors (Kwan, 2012; Alberini and Bigano, 2015; Dharshing, 2017; Ek and Söderholm, 2008; Neumann and Mehlkop, 2020; Wang et al., 2021; Sloot and Scheibehenne, 2022). A common type of interventions uses commitment strategies by asking participants to commit to specific energy-saving behaviors for motivating sustainable and pro-environmental behavior (Schwartz et al., 2015; Steinhorst and Klöckner, 2017; Xu et al., 2018; Rajapaksa et al., 2019; Ji et al., 2023; Kramer and Petzoldt, 2023).

Another type of intervention utilizes the idea of competition, where the participants with the best energy-saving performance are awarded. Such interventions have the advantage of engaging people in energy conservation and raise awareness about the connection between behavior change and energy use. However, it may be difficult to motivate individuals to stay in the competition (Konis et al., 2016; Bergquist et al., 2017; Nockur and Pfattheicher, 2020; Xie et al., 2021; Fijnheer et al., 2021).

The last type of intervention considered is collective or individual framing to influences people’s behavior and choices regarding sustainable initiatives. Framing interventions may utilize social comparison, goal setting, or commitment and are often evaluated in conjunction with the impact of social norms and norm-based strategies on long-term behavior. Hence, this type of interventions also depends on the individuals’ perception on and adherence to norms (Xu et al., 2015; Ghesla et al., 2020; Kacperski et al., 2023; Mäkivierikko et al., 2023; Syropoulos et al., 2023).

## 2 Materials and equipment

**Figure 1:**
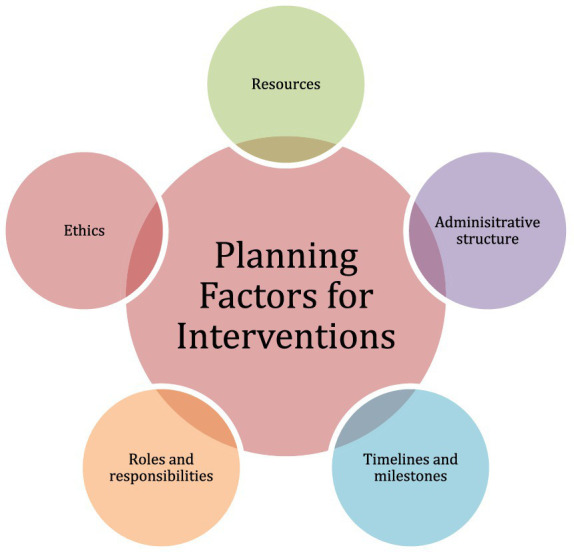
Planning factors for interventions.

The intervention guidelines rely on materials and equipment, or planning assets involving resources, timelines and milestones, administrative structure, roles and responsibilities, timelines and milestones, and ethics. Figure 1 demonstrates these factors.

### 2.1 Resources

Establishing the conformity of the intervention goals and objectives with the available and accessible resources in terms of personnel, equipment, and supplies is critical for successful implementation. Such endeavour involves a clear and SMART (specific, measurable, achievable, relevant, and time-bound) identification of the goals and objectives of each intervention package and matching these to the available resources, including personnel, equipment, materials, communication channels, and additional workforce. At this point, it is crucial to collaborate with partners and stakeholders to understand their unique needs, experiences, and constraints.

Critical considerations for resource planning are identifying priorities, collaborative planning, need assessment reporting, budget preparation, reporting, monitoring resources, considering resource efficiency, and establishing continuous communication. The allocation of resources according to the priority and importance of the goals and objectives amounts to considering the expected impact and the feasibility of achieving the goals with the available resources and allocating resources accordingly. Need assessment reporting refers to partners and stakeholders reporting the resources they need and how they plan to use them to implement the interventions. This includes details on the personnel or teams involved, the materials or equipment required, and any additional workforce that might be necessary. Collaborative planning is crucial to incorporate stakeholders’ past and current experiences and ensure that all stakeholders have a clear understanding of roles and responsibilities. Budgeting safeguards the resource-wise feasibility of the interventions by outlining the financial requirements associated with resource allocation. This budget considers personnel costs, material expenses, equipment procurement, and other related costs. Monitoring resources refers to the resources needed for continuous monitoring and adjusting if resource constraints or unexpected challenges arise. Considering resource efficiency amounts to tracking and evaluating the cost-effectiveness of the interventions. Regular communication aims to maintain open lines of communication with user partners throughout the intervention to address any resource-related issues and provide support as needed.

### 2.2 Administrative structure

Creating a clear administrative structure for each intervention is essential for ensuring that roles and responsibilities are well-defined, leading to efficient coordination and execution of interventions. Establishing a well-defined administrative structure enhances project management, minimises confusion, and facilitates more effective collaboration between users and scientific partners within the ENCHANT project. It is a critical component of successful intervention implementation. The key steps in establishing an administrative structure include defining job descriptions, identifying the staff responsible, the number of personnel and lines of responsibility, and succession planning.

Defining job descriptions starts with clearly defining the job descriptions for each role within the administrative structure, including the key responsibilities and tasks associated with each role, aligning with the objectives and requirements of the intervention plan. After that, the specific staff members responsible for each role within the administrative structure need to be determined. In doing so, the skills and expertise of the staff need to be matched with the tasks. The number of personnel involved in each role needs to be specified, considering the workload and complexity of tasks when determining the required staffing levels and project requirements. A complementary planning aspect is the definition of the lines of responsibility to establish reporting and communication channels, reporting schemes, and information flow. In anticipation of staff turnover and changes in responsibilities, a succession plan needs to be developed to ensure a smooth transition when staff changes occur. This may involve cross-training or identifying potential replacements in advance.

For the timely achievement of milestones, the administrative structure needs to be linked to the achievement of project milestones. Each role should have a clear connection to specific milestones to ensure that responsibilities are met within the project timeline.

### 2.3 Roles and responsibilities

The operational intervention plan should include the project partners’ and stakeholders’ roles and responsibilities. The partners are expected to define the specific roles and responsibilities as position descriptions for the personnel stating the required level of expertise in the related subject. Accordingly, the partners should address the competencies required and the qualifications needed. The roles and responsibilities must be clearly defined for an efficient management framework and governance structure.

The critical roles for each partner include a main responsibility (operational administrator), recruitment staff, staff responsible for the implementation and monitoring, data collection personnel, and data protection officer.

The operational administrator is responsible for examining the intervention progress periodically, executing corrective action where there is a failure to achieve tasks on time, ensuring that resources will be available when needed, and supervising, supporting and encouraging the staff to ensure that tasks are undertaken and reporting problems to other relevant partners.

The intervention recruitment staff establishes experiment and control groups by identifying potential research participants, delivering the informed consent procedures necessary for participation, and preparing templates of the Informed Consent Forms and Information Sheets covering voluntary participation and data protection issues in intelligible terms.

The staff responsible for the implementation and monitoring will organize the initiation, application and completion of the intervention by coordinating with other staff members regarding the activities in the intervention strategy and the timeline, ensuring the proper use of resources (human and/or material resources) for the achievement of the intervention’s goals, planning and organizing the method and timeline for how and when the resources will be used (e.g., in the case that posters or billboards are used for informative purposes, when, how and where these will be used), mitigating the effects of delays, interruptions and obstacles that are likely to pose barriers for efficient and routine-functioning of the intervention plans, managing the process and people/staff, ensuring the milestones are achieved, ensuring that all staff have the necessary information and resources to complete the tasks and discharge their responsibilities, communicating any changes to the relevant partners and stakeholders, ensuring that the ethical considerations are followed, and reviewing each member of personnel’s workloads and responsibilities.

The roles and responsibilities of the data collection personnel and data protection officer are signing and collation all necessary Informed Consent Forms before the collection of any data, securely storing the forms afterwards, ensuring required anonymisation is performed during data collection and processing, confirming that all data collection and processing will be carried out according to EU and national legislation, reporting the collected data for archiving, and providing detailed information for archiving on the procedures to be implemented for data collection, storage, protection, retention, and destruction, and confirmation that these procedures comply with national and other relevant legislations such as the EU legislation.

### 2.4 Timelines and milestones

Establishing a realistic and agreed-upon timeline with well-defined milestones is crucial for successfully implementing interventions. The key steps for developing and managing the timeline and milestones involve setting realistic timelines, collaborative agreements, specifying milestones, documenting milestones, assigning responsibilities, setting target dates, periodic tracking and reporting, flexible adaptation, communication, and recording milestone achievements.

Setting realistic timelines and estimating the time required to complete each project phase is significant. Factors such as recruitment, data collection, and implementation complexities need to be considered while avoiding setting overly ambitious or unattainable deadlines, as this can lead to frustration and potential setbacks. A collaborative agreement involving all partners and stakeholders in setting timelines and milestones is important as this provides a consensus on the proposed timeline, ensuring that all stakeholders understand the project’s timing. Specifying the measurable milestones, significant events or achievements that mark key progress points throughout the intervention process and documenting each milestone with clear descriptions of what should be achieved at each stage helps ensure that all stakeholders understand what is expected at each milestone. Once the timelines and milestones are agreed upon, assigning responsibilities for monitoring and achieving each milestone via identifying the key individuals or teams responsible for completing specific milestones ensures accountability and a focused effort to meet deadlines. Based on the timelines, target dates need to be set for each milestone. These dates should be based on a realistic assessment of each phase’s length and ensuring that target dates are aligned with the overall project timeline. Establishing the guidelines and a schedule for periodic tracking and reporting progress on milestones involves regular check-ins and updates, essential to monitor progress and identify any delays or issues early on. Project timelines may need to be adjusted through flexible adaptation as the project unfolds. This requires adapting and modifying timelines and milestones based on changing circumstances, challenges, or opportunities. As with all stages of the planning framework, effective communication is key to keeping all stakeholders informed about progress, challenges, and adjustments. Open and transparent communication ensures everyone is on the same page and can work collaboratively to address issues. Finally, recording milestone achievements by maintaining clear records of when each milestone is achieved helps track the project’s progress and can be valuable for evaluating the success of the intervention.

A standardised schema for timelines and milestones is crucial to any project, especially for more complex and collaborative projects. It facilitates coordination and progress tracking and allows for efficient communication among partners and stakeholders. Assessing and evaluating deviations from the timelines is essential for proactive problem-solving and managing unforeseen challenges. This practice helps identify critical issues early and find appropriate solutions, ultimately ensuring the successful implementation of interventions. The flexibility to adapt timelines and milestones in response to changing conditions or resource availability is a practical approach that can contribute to the overall effectiveness of the project. This standardised schema is a valuable tool for keeping the project on track and meeting its objectives.

### 2.5 Ethics

Ethical factors and compliance with national and international (such as European regulations) are crucial throughout the interventions. Data protection and informed consent need to be addressed, particularly in personal data collection. The interventions need to follow a comprehensive ethical framework to protect the rights and privacy of research participants while conducting interventions and data collection. This approach ensures that the research conducted is scientifically sound and respectful of ethical principles and legal requirements. The ethical framework for interventions includes concerns regarding recruitment, informed consent procedures, data collection and processing activities, data storage and curation, and anonymisation.

Concerning recruitment, voluntary participation and opt-out need to be secured. The interventions should target adult participants who can provide informed consent. Participants need to be informed about the study’s purpose, data collection procedures, and the potential usage of data, such as energy consumption data. Participants need to have the right to withdraw from the study without facing any penalties or providing a reason. A contact channel (phone and email) needs to be provided for participants to inquire about their stored data. All information needs to be presented in written form during the recruitment process, and informed consent forms need to be signed before any data collection activity.

Written informed consent needs to be sought from participants wherever possible; explicit online consent can be used for online surveys. Informed consent needs to comply with forms provided by relevant authorities and organisations. Templates for consent forms and information sheets must be prepared in national languages when necessary and archived.

Ethical data collection principles must be addressed to ensure quality, accuracy, efficiency, effectiveness, feasibility, and security. Procedures need to be developed to meet general scientific quality criteria, and data must be correct, complete, and reliably entered. Confidentiality and data protection need to be ensured in line with legal requirements.

Personal data needs to be stored in safe storage spaces, and periodic backup routines need to be implemented to prevent data loss.

Data Anonymization is a crucial practice to protect the privacy of stakeholders and users. Personally identifiable data need to be anonymised and aggregated. The mapping between anonymised and actual IDs must be safeguarded and accessible only to those directly working with the data.

## 3 Methods

The guidelines and application principles for the interventions cover the preliminary work and actual implementation, along with a phased timeline for monitoring and follow-up. Among the objectives of these guidelines are to ensure appropriate timeline management, resource allocation, and operational planning to maximise the likelihood of achieving the desired impact, as well as collecting the data for analysing the intervention results and enhancing the replicability and reproducibility of interventions.

As part of the process, the guidelines and operational plan for the interventions have been developed using a framework that addresses key issues of autonomy, information exchange, governance, and clearly defined responsibilities. The Guidelines and Operational Intervention Plan begins by selecting intervention types for each user-partner. It then proceeds through a multi-staged planning framework, with defined milestones for each stage. This framework provides a structured approach to ensure the interventions are effectively designed and implemented.

### 3.1 The process for designing the guidelines and the planning framework

The guidelines are prepared considering a comprehensive framework, including data analysis, impact assessment, tool creation, and effective dissemination of results to various stakeholders. The work for preparing the guidelines is supported by different pre-intervention work packages involving methodology design, ethics, data management, intervention design, protocol design, and pilot interventions, and post-intervention work packages involving tools for replicability, suitability for upscaling, and comparability, impact assessment and evaluation.

The planning framework for the efficient and effective implementation of interventions involves three main phases, each corresponding to one stage in the timeline of the systematic planning process. These phases are, Planning and Design, Intervention Implementation, and Analysis and Reporting.

**Figure 2:**
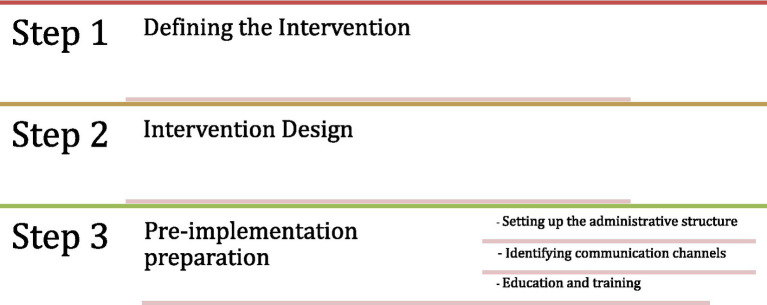
Phase I: planning and design.

Phase 1—Planning and Design refers to defining the intervention, focusing on the current situation, needs, and concerns. Understanding the specific characteristics, needs, and concerns of the target population and documenting the current situation is crucial. This information influences the intervention strategy. The intervention design then takes into consideration the analysis of the current situation and designs the interventions to maximise changes in energy-related behaviors while suiting the current situation of the relevant society. Significant considerations at this stage include implementability, replicability, and upscalability. Following the intervention design, the next step is the pre-implementation preparation. This step focuses on the practical aspects that should be considered before the intervention kick-off, such as setting up the administrative structure, assigning roles and responsibilities, educating and training personnel, and identifying necessary communication channels and equipment. Figure 2 provides an overview of the first phase.

Following the planning and design phase, the second phase is the Intervention Implementation Phase. Phase II is the most extensive phase and involves a wide spectrum of stages to ensure the success of the interventions. To begin with, the Intervention Implementation Phase involves establishing effective communication with the project coordinator and implementation partners, which is particularly essential for coordination and progress monitoring as well as for smooth progress and identifying solutions to potential challenges. A critical component of Phase II is setting a timeline and constructing a standardised reporting structure. This stage involves specifying timelines and milestones, which are crucial for tracking progress and maintaining the project schedule since standardised reporting enhances the measurement of success.

The following stage is the recruitment stage, where participants are selected and recruited, informed consent is secured, and the purpose of the study is explained. The recruitment stage is complemented by randomisation and pre-intervention data collection stages. In the randomisation stage, participants are randomly assigned to experimental and control groups. This is vital to maintain methodological rigour and prevent self-selection bias. In the pre-intervention data collection stage, data is collected from participants before the intervention begins to establish baseline information.

**Figure 3:**
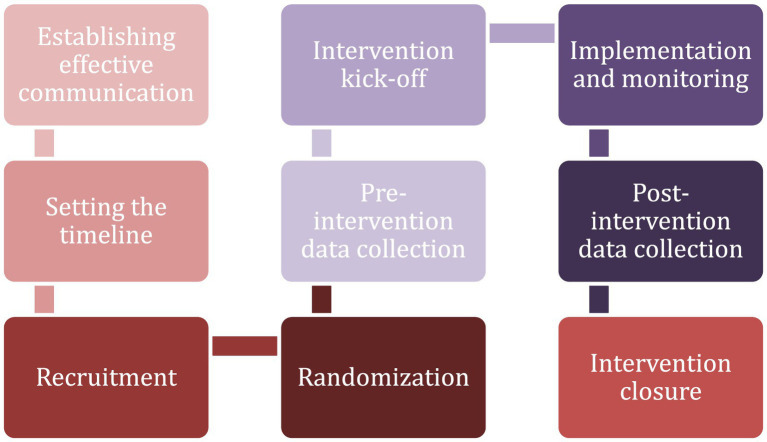
Phase II: intervention implementation.

The next stages of the Intervention Implementation Phase include intervention kick-off, intervention implementation and monitoring, post-intervention data collection, and intervention closure. The intervention kick-off is conducted through the designated communication channels after ensuring that the preceding planning steps are completed. Intervention implementation and monitoring keep track of the intervention progress and milestones. This stage also involves identifying deviations from the plan, developing measures to recover the intervention progress, and adapting to unexpected events during intervention implementation. Post-intervention data collection is as important as the intervention implementation since the performance of the intervention cannot be assessed without collecting the post-intervention data and comparing it with the pre-intervention data to identify the behavioral changes resulting from the intervention. The final stage of the Intervention Implementation Phase is intervention closure, which involves reaching milestones, completing relevant tasks, and communicating with relevant stakeholders about the completion of the intervention. Figure 3 provides an overview of Phase II.

**Figure 4:**
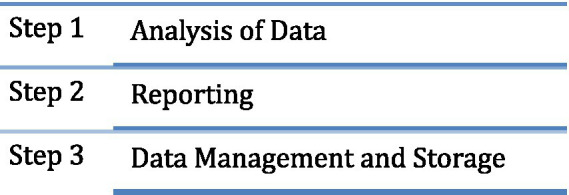
Phase III: analysis and reporting.

The third phase of planning is the Analysis and Reporting phase. This phase involves the analysis of the data collected during the pre-intervention, intervention, and post-intervention stages, including primary and secondary data. An essential aspect to handle at this step is the anonymisation of data to comply with privacy and ethical considerations. Following the analysis of the intervention data, the intervention needs to be reported transparently to demonstrate the quality of the study and allow for verification by others. The final stage of the Analysis and Reporting Phase is the commonly overlooked data management and storage stage. The guidelines aim to ensure that interventions are designed, implemented, and reported transparently and systematically, allowing for verification and further research. Data management and storage principles include recording and securely holding data with metadata, backing up electronic data, and ensuring digital continuity and future accessibility. These principles are crucial to safeguard data security and accessibility while promoting future research opportunities. These stages are demonstrated in Figure 4.

Table 1 provides a checklist that can be adapted for a particular intervention and utilised to keep track of the operational steps of interventions.

**Table 1:** Intervention guideline checklist.

Step	Action	Date planned for completion	Actual completion date	Deviation(s)	Corrective measures
1	Define the intervention				
2	Design the intervention				
3	Setup administrative structure				
4	Assign roles and responsibilities				
5	Education and training				
6	Identify communication channels				
7	Set the timeline				
8	Recruitment				
9	Randomization				
10	Pre-intervention data collection				
11	Intervention closure				
12	Analysis of data				
13	Reporting				

## 4 Results

For achieving the desired results from the interventions, other key factors that need to be considered include operational monitoring mechanisms, proper reporting of the intervention process, and *a priori* consideration of the challenges and barriers.

### 4.1 Operational monitoring mechanisms

A methodological approach is needed for operational monitoring mechanisms for interventions to ensure efficient oversight of the intervention implementation process. Based on the process control techniques (Pourmirza et al., 2017; Jakhar, 2017; Cangussu et al., 2003), operational monitoring mechanisms aim to track, assess, and measure the actual performance of interventions in comparison to the expected mode of operation. The key steps and processes involved are defining the planned/expected mode of operation, tracking the implementation, assessing/measuring the actual performance, comparing the actual performance with the expected mode of operation and identifying the differences/deviations from the expected mode of operation, and determining reasons for differences/deviations from the expected mode of operation and taking actions for restoring the expected mode of operation.

Defining the planned/expected mode of operation refers to analysing the intervention designs and planning guidelines for establishing intervention timelines, identifying data requirements for key performance indicators (KPIs), and considering compliance with ethical and data protection requirements.

Tracking the Implementation process through the key stages, including recruitment, randomisation, pre-intervention data collection, intervention kick-off, execution of the intervention, post-intervention data collection, and closure, is central to intervention monitoring. This can be efficiently done using an intervention monitoring checklist and an intervention timeline Gantt Chart to track and monitor the timely execution of the intervention stages. A sample intervention monitoring checklist that can be adapted for particular interventions is demonstrated in Table 1 in the Appendix.

For assessing or measuring the actual performance of the intervention, KPIs on the progress and outcomes at each stage are utilised, monitoring actual numbers, timelines, and data collection procedures and validating data collection processes. A comparison is made between actual performance and the planned mode of operation. For this purpose, both qualitative and quantitative assessments are included. In case of differences or deviations from the planned mode of action, stakeholders are consulted to assess the impact of deviations and actions are planned and implemented to restore the expected mode of operation and mitigate impacts.

The monitoring framework is implemented periodically to ensure interventions progress as planned, where the monitoring frequency is determined based on the specific intervention’s needs. The monitoring framework allows for periodic and additional monitoring, depending on the feedback and issues raised during the implementation. This approach ensures that deviations and challenges are promptly addressed, data collection processes remain robust, and interventions are aligned with their expected outcomes.

The methodology provides a structured approach to intervention monitoring. It allows for flexibility in addressing unforeseen issues, such as the impact of the COVID-19 pandemic, which may affect the interventions’ progress and results.

### 4.2 Reporting

The framework for intervention reporting follows a structured approach for documenting and analysing the project’s interventions. This framework provides a comprehensive structure for reporting on interventions to ensure transparency and clarity. This structured framework ensures that intervention reporting provides a clear, detailed, and comprehensive account of each intervention’s context, implementation, methodology, outcomes, and implications. This systematic approach helps the project analyse and document its interventions effectively. The key sections included in the framework are as follows:

- Title of the implementation and brief summary: introduction, introduction: background, problem definition, intervention definition, targeted behavioral change, implementation strategy, methods, and expected goals.
- Study design: setting, participants, variables, data sources and type of data, and ethical considerations.
- Analyses: descriptive data, outcomes, and main results.
- Discussion: key results, limitations, interpretation and alignment with prior research, contextual factors, and generalizability.
- Concluding remarks regarding the intervention.

### 4.3 Addressing potential challenges and barriers

Implementing interventions for behavioral change, especially in the context of sustainability and energy efficiency, can pose various challenges and barriers.

To address these challenges and barriers, it’s essential to have a flexible and adaptive approach, regularly monitor progress, and be prepared to make adjustments when necessary. Close collaboration between scientific partners and user partners can help ensure that interventions are designed and implemented effectively, considering local conditions and requirements. Additionally, ongoing communication with participants and stakeholders is crucial to maintaining engagement and addressing concerns as they arise.

**Figure 5:**
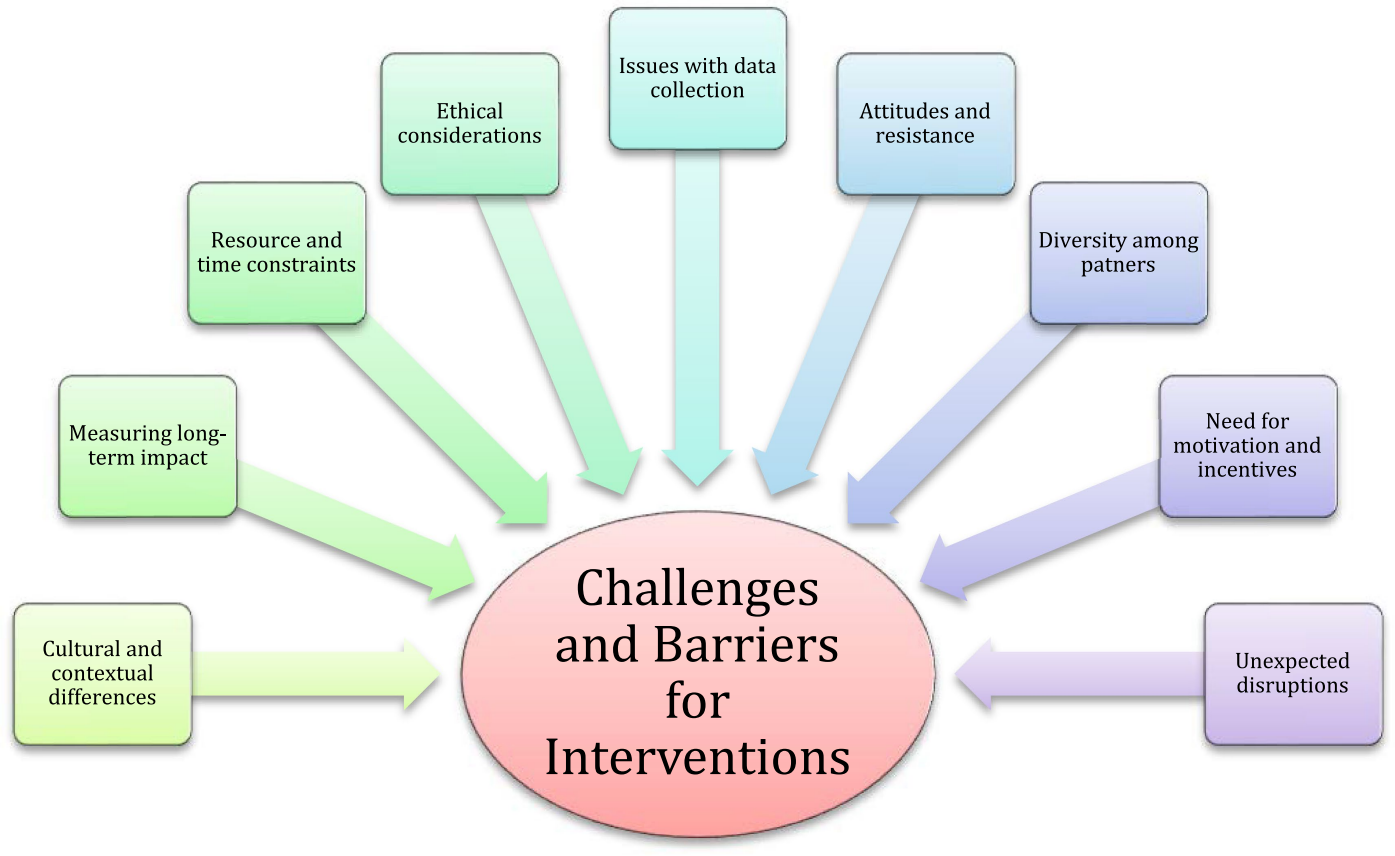
Potential challenges and barriers to interventions.

The potential challenges and risks are depicted in Figure 5 and explained.

#### 4.3.1 Achieving target population numbers

Meeting the predefined target population numbers can be challenging, especially if the intended audience is not readily accessible. Identifying and recruiting the right participants can be time-consuming and require considerable effort. In order to overcome such challenges, local organizations can be collaborated with, and their networks can be utilized for recruitment. The recruitment process can also benefit from social media and online platforms.

#### 4.3.2 Impact of unexpected disruptions such as the COVID-19 pandemic

Disruptions such as the COVID-19 pandemic may affect participation levels, making it harder to reach the desired numbers and impacts. Restrictions and concerns related to the pandemic could limit people’s willingness to participate in interventions. Concerning this challenge, contingency plans including flexible participation options and remote communication tools for the interventions may be effective.

#### 4.3.3 Need for motivation and incentives

Finding effective strategies to motivate and incentivise households can be challenging. People have diverse motivations and may respond differently to various incentives. Developing a standardised approach that works across different partners and samples can be complex. The referral system and a well-designed set of incentives (such as small gifts over time, with progress, and gamification of the interventions) can be used to address this challenge.

#### 4.3.4 Diversity among partners

Implementing similar interventions by different partners with their unique samples and contexts can lead to diversity or even conflicts in the intervention strategies. Standardisation is essential, but it should also allow flexibility to adapt to local needs. Utilizing common communication channels through which participants feel comfortable to share their ideas can help alleviate this challenge. Moreover, regular meetings with participants also serves to achieve a standard and shared intervention perspective.

#### 4.3.5 Attitudes and resistance

Households’ attitudes, resistance, or negative reactions to interventions can hinder their success. Addressing resistance and concerns related to privacy and data security is crucial. Effective communication and engagement strategies are essential to mitigate these challenges. Transparent communication, explicit addressing and discussion of resistance and negative reactions are helpful regarding this challenge. In some instances, local leaders can act as mediators and encourage participation in the intervention.

#### 4.3.6 Data collection

Collecting and post-intervention data accurately and consistently can be challenging. It’s important to ensure that data collection methods align with the intervention design and follow the guidelines closely. Adherence to the research design and the guidelines is important for addressing this challenge.

#### 4.3.7 Ethical considerations

Ethical considerations, including informed consent and data privacy, are paramount. Ensuring that participants fully understand the interventions, their implications, and how their data will be used is essential to maintain trust and minimise ethical concerns. To this end, periodic ethical reviews can also be utilized.

#### 4.3.8 Resource and time constraints

Limited resources in terms of personnel and funding can affect the scale and scope of interventions. Ensuring that interventions are realistic within the available resources is vital. A proper intervention planning and well-designed guidelines help in addressing this challenge.

#### 4.3.9 Measuring long-term impact

Determining the long-term impact of interventions can be challenging, as behavior change may take time to manifest. Measuring sustained energy behavior and lifestyle changes requires extended follow-up and data collection. Regarding this challenge, post-intervention surveys and similar long-term monitoring tools can be utilized.

#### 4.3.10 Cultural and contextual differences

Implementing interventions across diverse geographical, cultural, and organisational contexts may require adaptation to local norms and preferences. What works in one region may not work in another. Collaborating with local experts and adopting intervention messages and the general communication with respect to local values, beliefs, and norms may be useful.

## 5 Discussion

Projects and similar endeavours aiming for behavioral change towards environmentally friendly lifestyles implement a range of behavioral interventions to achieve them. The main objectives for these interventions are increasing the sustainability of the households through promoting more sustainable energy choices and lifestyles that aim at changes in energy behavior under real-life conditions and reviewing and systematising knowledge related to interventions for influencing human behaviors towards more sustainable energy choices.

An analysis of the literature points to gaps in terms of the existence of well-defined methods for interventions. Hence, a systematic set of guidelines have the potential to contribute to the replicability of the interventions, and the reliability of their findings. As described in the manuscript, the guidelines and the established monitoring mechanisms also enhance a better understanding of the underlying processes of the interventions. Following the guidelines also enhances the proper allocation of resources and reducing the costs of the interventions.

The interventions’ design, planning, implementation, and monitoring need to be conducted systematically. A systematic framework is essential to contribute to the design, execution, and supervision of the interventions, provide standardisation and enhance collaboration with the intervention partners. In the case of a wide geographical coverage and the involvement of multiple stakeholders, intervention guidelines are crucial in ensuring that the interventions are well-coordinated and adhere to predetermined timelines.

A well-designed administrative structure with clearly defined roles is essential for successfully implementing the interventions. This structure helps ensure that the responsible staff members understand their roles and responsibilities, which is crucial for the smooth execution of the project. Ethical considerations are an indispensable component of behavioral interventions. Ethical issues can arise at any stage of the interventions, from recruitment and informed consent procedures to data collection, anonymisation of data, and data storage. Addressing ethical concerns appropriately is vital to protect the interests of participants and partners, maintain the validity of results, and ensure the project’s overall success. Ethical requirements are incorporated into the guidelines and operational plan to guide ethical decision-making throughout the project.

Monitoring the interventions is critical due to the varied nature of the intervention designs and the project’s wide geographical scope. The monitoring serves multiple important purposes, such as coordination, assessing validity, data collection and processing, and ethics and data management. Systematic monitoring is necessary to establish coordination between and within the interventions, ensuring that various activities align with the project objectives. Monitoring the progress of the interventions also allows for assessing the validity of the results and conclusions drawn. Corrective actions can be taken promptly if any issues affecting the validity arise. Collection and processing of pre-intervention and post-intervention data are crucial for the intervention impact. Therefore, monitoring ensures that data is collected and utilised following the intervention designs. Ethical requirements, data privacy, and data management are essential considerations throughout the project. Monitoring helps ensure that recruitment, informed consent, data collection, anonymisation, and data storage adhere to these requirements.

The methodology for the intervention monitoring plan is adapted from general control process methodologies (Wright, 2023; Lumen Learning). It involves the key step of defining the expected mode of operation, tracking the implementation, assessing/measuring the actual performance, and comparing actual performance with the expected mode of operation: actual performance is compared with the planned mode of operation to identify any differences or deviations, determining reasons for differences/deviations, and taking actions for restoration. The utilization of control process methodology aims better resource allocation, fast adaptability, informed decision-making, and strategy adaptation.

Although the guidelines presented in this manuscript originate from the energy efficiency domain, they are applicable to environmental behavior change interventions in general.

The monitoring process is ongoing and needs to be repeated periodically. It also needs to be flexible, allowing for additional intermediate monitoring iterations as required. This adaptability ensures that the interventions remain on track and that any unforeseen issues can be addressed promptly. Regular updates are made to the planned/expected mode of operation based on the monitoring results, allowing for continuous improvement and optimisation of the interventions.

## Data Availability

Publicly available datasets were analyzed in this study. This data can be found at: https://zenodo.org/records/10580655, https://zenodo.org/records/10579929, https://zenodo.org/records/10590722, https://zenodo.org/records/10591860, https://zenodo.org/records/10591860, https://zenodo.org/records/10580194, and https://zenodo.org/records/10591320.

## Appendix

**Table A1:** Sample intervention monitoring checklist template.

Partners: Intervention: Date:													
	Pre-intervention					Implementation			Post-intervention				
	Recruitment	Randomization		Pre-intervention data collection		Intervention kick-off		Implementation of the intervention	Post-intervention data collection			Intervention Closure	
	Targeted number of participants vs actual recruited are compared	Experimental group the intervention is implemented is formed	A control group without any intervention is formed	The data collection method is planned	Designed data to be collected vs. actual data collected are compared	Intervention is started on time	Participants and involved parties are informed about the procedures	Guidelines and Operational Plan followed according to the intervention designs and RCT research protocol and the timelines	The data collection method is as planned and is confirmed	Planned data to be collected vs. actual data collected are compared	Compatibility with pre-intervention data is checked	Intervention is closed on time	Any delay/deviations affecting intervention closure are checked
Deviation from plan													
Ethical considerations													
